# An overview of occupational noise-induced hearing loss among workers: epidemiology, pathogenesis, and preventive measures

**DOI:** 10.1186/s12199-020-00906-0

**Published:** 2020-10-31

**Authors:** Kou-Huang Chen, Shih-Bin Su, Kow-Tong Chen

**Affiliations:** 1 School of Mechanical and Electronic Engineering, Sanming University, Sanming, 365 Fujian Province China; 2 Department of Occupational Medicine, Chi-Mei Medical Center, Tainan, 710 Taiwan; 3 Department of Occupational Medicine, Tainan Municipal Hospital (managed by Show Chwan Medical Care Corporation), No. 670, Chongde Road, East District, Tainan, 701 Taiwan; 4 Department of Public Health, College of Medicine, National Cheng Kung University, Tainan, 701 Taiwan

**Keywords:** Noise-induced hearing loss, Occupational diseases, Prevalence, Pathogenesis, Preventive measures

## Abstract

Occupational noise-induced hearing loss (ONIHL) is the most prevalent occupational disease in the world. The goal of this study was to review the epidemiology, pathogenesis, and preventive measures of ONIHL among workers and provide evidence for the implementation of control measures. Literature studies were identified from the MEDLINE, PubMed, Embase, Web of Science, and Google Scholar using the search terms “noise-induced hearing loss” “prevalence”, “pathogenesis”, and “preventive measures”. The articles reviewed in this report were limited from 2000 to 2020. Articles that were not published in the English language, manuscripts without an abstract, and opinion articles were excluded. After a preliminary screening, all of the articles were reviewed and synthesized to provide an overview of the current status of ONIHL among workers. The mechanism of ONIHL among workers is a complex interaction between environmental and host factors (both genetic and acquired factors). The outcomes of noise exposure are different among individual subjects. Clinical trials are currently underway to evaluate the treatment effect of antioxidants on ONIHL. Noise exposure may contribute to temporary or permanent threshold shifts; however, even temporary threshold shifts may predispose an individual to eventual permanent hearing loss. Noise prevention programs are an important preventive measure in reducing the morbidity of ONIHL among workers.

## Introduction

Hearing loss due to noise exposure in the workplace is a significant health problem worldwide [1, 2]. Occupational noise-induced hearing loss (ONIHL) is a common occupational disease. Since the eighteenth century, reports have noted that copper miners experienced hearing loss due to the noise exposure from hammering on metal [3, 4]. It is estimated that 1.3 billion people suffer from hearing loss due to noise exposure [3]. Worldwide, occupational noise exposure is responsible for 16% of cases of disabling hearing loss in adults [1, 3, 5]. This indicates that ONIHL does not directly cause premature mortality but does result in substantial disability.

The impacts of occupational noise exposure cause a tremendous financial and disease burden on both individual and society. In the USA, it is estimated that the annual compensation for ONIHL is approximately $242.4 million [3, 6]. This economic burden on society is extremely high and continually increasing. Previous studies have indicated that workers employed in the construction, manufacturing, mining, agriculture, utility, and transportation, industries, military personnel, and musicians have the highest risk for ONIHL [7–9].

ONIHL can limit an individual’s ability to communicate with others and can lead to increased social stress, sadness, diminish confidence, poor self-identity, and bad interpersonal relationships [6, 9]. Commonly, hearing loss interferes with communication and can hinder personal attention and cognition [6, 9]. Older people with mild hearing loss have a twofold increased risk of dementia, whereas those with severe hearing loss have a fivefold increased risk of dementia [10]. ONIHL is a complex and preventable disease [11, 12]. Understanding the mechanism of ONIHL and distribution of those affected by ONIHL is important to develop proper preventive measures. Therefore, the aim of this study was to explore the prevalence, pathogenesis, and preventive measures of ONIHL.

## Methods

In preparing this review, we searched several online databases, including MEDLINE (National Library of Medicine, Bethesda, Maryland, USA), PubMed, Embase, Web of Science, and Google Scholar for relevant published papers using a combination of the following keywords “noise-induced hearing loss”, “prevalence”, “pathogenesis”, and “preventive measures”. The references listed in the retrieved studies were also searched in an attempt to find additional relevant studies. The steps of the review process followed the preferred reporting items for systematic reviews and meta-analysis (PRISMA) guidelines. The inclusion criteria of papers for reviewing were exposure to occupational noise alone or in combination with other factors, hearing loss and other health outcomes, and the statistical association between occupational noise and hearing loss/other health outcomes. We screened all reference lists of relevant studies in order to identify any missing publications. The literature review was completed in March 2020.

## Results and discussion

**Fig. 1:**
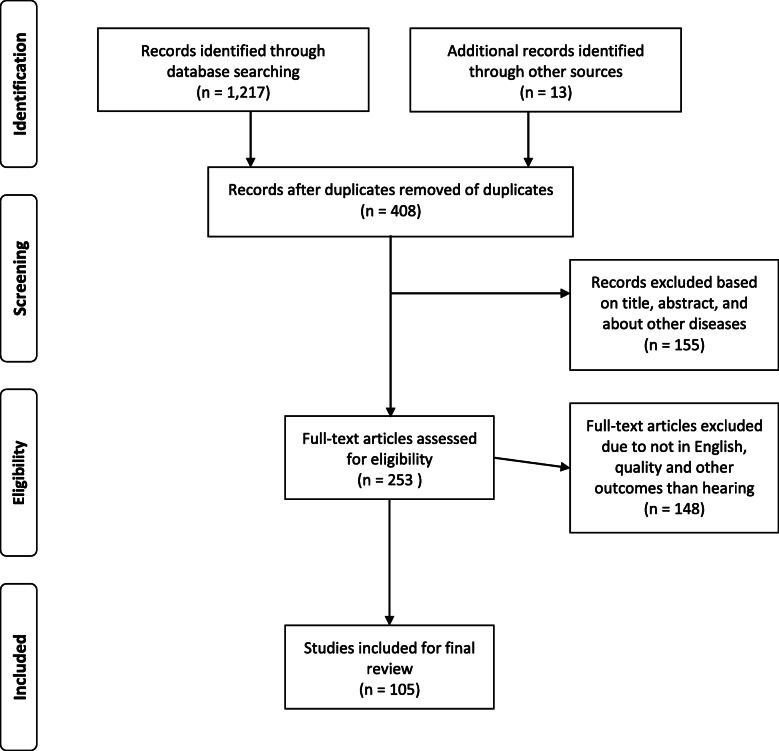
Flow diagram for the literature research in the study

A total of 1230 documents were retrieved from the abovementioned sources. All titles and abstracts from the literature search were assessed by two investigators against the inclusion criteria for possible relevance independently. We solved any discrepancies through consensus. References that we judged to be potentially relevant were read in full text and evaluated. Relevant original studies were quality-assessed by one of the investigators using a checklist developed by Hoogendoorn et al. [13] for the evaluation of observational studies. We defined high quality as a score of more than 50% on the internal validity scale of the checklist. The articles reviewed in this report were limited from 2000 to 2020. Articles that were not published in the English language, manuscripts without an abstract, and opinion articles were excluded. Finally, a total of 105 documents were considered for review (Fig. 1).

After the articles were selected, we extracted potential information related to epidemiology, pathogenesis, and preventive measures and classified the information accordingly.

### Defining ONIHL and related information

ONIHL is defined as a partial or complete hearing loss in one or both ears as the result of one’s employment; it is a function of continuous or intermittent noise exposure and usually develops slowly over several years [14]. ONIHL is different from the acoustic trauma, which is characterized by a sudden change in hearing as a result of a single exposure to a sudden burst of sound, such as explosive blasts, gunfire, and attendance at loud concerts [14, 15]. Although there is increasing evidence that asymmetrical hearing loss occurs among individuals with ONIHL [16], typically, ONIHL is a sensory neural phenomenon involving injury to the inner ear. It is bilateral and symmetrical, usually affecting the higher frequencies at 4 kHz, with spread to neighboring frequencies of 3 and 6 kHz, and some hearing recovery at 8 kHz [14, 17]. With further noise exposure, the hearing loss can involve the lower frequencies at 0.5, 1, or 2 kHz [4]. ONIHL is proposed to be on average no higher than 75 dB in the high frequencies and no higher than 40 dB in the lower frequencies [18]. Four grades of hearing impairment are classified by the WHO: slight (audiometric ISO value 20-40 dB), moderate (41-60 dB), severe (61-80 dB), and profound (81 dB or greater) [14, 19]. The audiometric ISO values are averages of values at 0.5, 1, 2, and 4 kHz. Self-reported hearing problems and physical examinations are sometimes used for the detection of ONIHL in the workplace [20].

The Occupational Safety and Health Administration (OSHA) and the National Institute for Occupational Safety and Health (NIOSH) provided the standard exposure levels of noise [21–23]. For OSHA [21], the permissible exposure limit (PEL) in the occupational noise exposure standard (29 CFR 1910.95) is 90 dBA, for an 8-h time-weighted average (TWA) with a 5-dB exchange rate (increase or decrease in dB corresponding to doubling or halving the noise dose). An action level of 85 dBA, for an 8-h TWA (50% of PEL) was added in the 1983 Hearing Conservation Amendment (HCA), in which a hearing conservation program must be made available to workers whose exposures equal or exceed the action level. In addition, an 80-dB threshold level in the computation of noise dose was proposed in the OSHA-HCA criteria. By including sound energy doses from 80-90 dBA, the noise dose measured under the OSHA-HCA criteria is typically higher than the corresponding compliance measurement.

For NIOSH, an 8-h time-weighted average exposure limit of 85 dBA and a 5-dB exchange rate were recommended in the 1972 Criteria for a Recommended Standard: Occupational Exposure to Noise [22]. These criteria were further revised in 1998. The recommended exposure limit (REL) remains at 85 dBA, but the 5-dB exchange rate has been replaced with a 3-dB exchange rate. Therefore, under this requirement of an exchange rate of 3-dB, a 4-h of exposure at 88 dBA is as equally hazardous as 8 h at 85 dBA. Furthermore, all workers exposed to noise levels above the REL are recommended to be enrolled in a hearing loss prevention program (HLPP) [22, 23]. The main difference between the NIOSH criteria and the OSHA-HCA criteria is that the former used 85 dBA with a 3-dB exchange rate for noise exposure measurements, whereas the latter uses 90 dBA with a 5-dB exchange rate [24]. A previous study showed that when the NIOSH criteria were adopted as an OSHA standard, there was a 2.7-fold increase in the number of workers enrolled in a hearing prevention program [24].

The International Organization for Standardization provided criteria for safe noise levels in ISO-1999 [25]. The ISO-1999 standard of noise exposure is based on data collected during the 1950s and 1960s [26]. The studies used to prepare the ISO-1999 standard mainly considered steady-state noise exposure [27]. The safe doses of noise exposure required in this standard assume that hearing loss is a function of total exposure ignoring the effects of the temporal characteristics of noise and complex noise environments [28]. Accordingly, this standard provides lower estimates of the risk of noise-induced hearing loss (NIHL). To solve this problem, a new noise metric with a temporal correction term was designed. A previous study showed that the kurtosis correction term generally improves the correlations of metrics with NIHL [29]. Furthermore, the current NIOSH guidelines suggest that a 140-dB sound pressure level (SPL) limit should be used for impulsive noise, whereas an 85 dBA permissible exposure level (PEL) with a 3-dB exchange rule should be used for complex noise [21]. Recent studies have shown that SPL combined with a kurtosis correction term may serve as a good noise metric for the assessment of ONIHL risk [29, 30].

### Epidemiology

The morbidity of ONIHL has been estimated for different countries. The criteria for ONIHL among workers vary from country to country [6–9]. The burden associated with occupational noise varies from 11.2% (South Africa) to 58% (USA) (Table 1) [31–65]. Overall, the prevalence of ONIHL is generally higher in the less developed regions of the world [3].

**Table 1:** A summary of the prevalence of noise-induced hearing loss among workers in the world by year reported by occupation by country, 2009-2019

Year of reported	Countries/regions	Population/exposure/sex/age	Prevalence (hearing threshold)	Reference
2009	French	Policemen (*N* = 887) Mean age: 37.6 years	28% (≧ 30 dB)	[31]
		Civil servants (*N* = 805) Mean age: 41.8 years	16% (≧ 30 dB)	
2010	Norway	General population (*N* = 49,948) Mean age: 48.0 years (20-101)	10.3% (≧ 35 dB)	[32]
2011	Dutch	Construction/male workers (*N* = 24,670) Mean age: 44.3 years	22.1% (≧ 40 dB)	[33]
2012	Ghana	Stone crushing workers (*N* = 140; male: 137, female: 3) Mean age: 42.6 years	21.5% (≧ 34 dB)	[34]
2012	South Africa	Gold miners (underground noise exposure; *N* = 33,749)	11.2% (≧ 40 dB)	[35]
		Gold miners (surface noise exposure; *N* = 7456)	14.1% (≧ 40 dB)	
2013	Zimbabwe	Mining workers (*N* = 169; male = 158, female = 11) Mean age: 34.8 years	37% (≧ 40 dB)	[36]
2014	Ghana	Market mill workers (*N* = 101)	24.8% (≧ 25 dB)	[37]
2015	Nepal	Carpenters/male (*N* = 88) Median age: 23 years (20-31)	31% (≧ 50 dB)	[38]
		Sawyers/male (*N* = 36) Median age: 30 years (20-45)	44% (≧ 50 dB)	
2016	USA	Mining workers (*N* = 7398; male: 7895, female: 3) Median age: 38.7 years (18-75)	24% (≧ 40 dB)	[39]
		Oil and gas extraction (*N* = 1072; male: 977, female: 95) Median age: 36.0 years (16-79)	14% (≧ 40 dB)	
2018	USA	AFFH sector (*N* = 17,290; male: 12,482, female: 4808) Media age: 34.9 years (18-75)	15.0% (≧ 40 dB)	[40]
2018	USA	Construction workers/male (97%)/Caucasian (89%) (total *N* = 19,127) Mean age = 59.2 years	58% (≧ 40 dB)	[41]
2019	South Thailand	Sawmill workers (*N* = 699; male: 335, female: 364) Mean age: 33.5 years (± 10.2)	22.8% (≧ 25 dB)	[42]
2019	China	Automotive manufacturing (*N* = 6667; male: 6427, female: 240)	28.8% (≧ 30 dB)	[43]

*AFFH* agriculture, forestry, fishing, hunting; *IQR* interquartile range; *OGE* oil and gas extraction

Previous studies have shown that males experience more effects after exposure to occupational noise than females [1, 66]. This may be due to males usually having greater exposure to noise at work than females due to differences in occupational categories, economic sectors of employment, and lifetime work history. Another possible reason is the hormone-driven physiological differences between sexes. Several animal and human studies have demonstrated that women may be protected against hearing loss because of estrogen and its signaling pathways [66].

The age groups from 30-44 and 45-59 years are at higher risk when exposed to occupational noise, corresponding to the ages of peak labor force participation [67]. A previous study showed that the affected fraction decreased by age group after 30-44 years old [1], indicating the heavy impact of occupational noise on the burden of hearing loss at younger ages [1–3]. If people suffering from hearing loss at a younger age have a longer duration of disability, then more years of disability contribute to the disability-adjusted life year (DALY) calculation.

It has been reported that ONIHL among workers has been significantly associated with an increased risk of work-related injuries [68]. The suggested reasons for this finding are that higher noise levels obstruct the ability to hear warning signals, monitor equipment, react to environmental sounds, and coordinate with other workers. The burden of hearing loss among noise-exposed workers varies by industry and occupation. Overall, the industries at the highest risk for hearing loss are the mining, textile, buildings construction, and wood product manufacturing sectors [31–65].

In addition to auditory outcomes, ONIHL has also been associated with a number of nonauditory sequelae. Bad mood, poor cognition, sleep disorders, and cardiovascular diseases are the frequent complications of ONIHL [6]. Previous studies have shown that higher levels of noise exposure are associated with higher morbidity and mortality of cardiovascular disease [69–74]. It is estimated that people with hearing loss have 10-20% excess mortality [75]. It has been suggested that noise exposure induces reaction of the autonomic nervous system and endocrine system, leading to increased secretion of the stress hormone, which in turn may lead to an increased risk of hypertension, coronary heart disease, and stroke [76, 77].

### Pathogenesis

The burden of hearing loss related to the specified noise exposure is variable. Similar to the processes of other diseases, the pathogenesis of ONIHL shows a complex interaction between genetic and environmental factors. It has been reported that up to 50% of individual variations in people with ONIHL may be associated with hereditary factors [36]. In addition, individual demographic factors such as age, preexisting sensorineural hearing loss, chronic diseases (e.g., hypertension and diabetes mellitus), history of smoking, and use of ototoxic medications may influence the degree of damage to the inner ear caused by noise injury [5].

The human auditory system is composed of the outer ear, middle ear, and inner ear. The outer ear gathers sound energy and transmits it to the middle ear through the ear canal and the tympanic membrane and then transmits the vibrations to the inner ear through the three tiny bones (malleus, incus, stapes) in the middle ear [4]. These vibrations are conveyed to the cochlea, where they generate pressure to vibrate the basilar membrane, where hair cells convert it to action potentials [78]. Mechanical coupling causes sound energy to be efficiently transmitted from the air to the cochlea. A previous study indicated that hair cell electrical responses transduce sound via submicrometer deflections of hair bundles, which are arrays of interconnected stereocilia containing mechanotransducer (MT) channels [78]. Activation of MT channels is initiated by tension in extracellular tip links bridging adjacent stereocilia, and they can respond within microseconds to nanometer displacements of the bundle, facilitated by several steps of Ca^2+^-dependent adaptation. The vibrations are converted by the inner hair cells into electrical impulses, and the impulses are delivered to the brain through the auditory nerve [4, 30]. The inner ear has two parts, the vestibular system and the cochlea. The organ of Corti, a specialized sensory epithelium resting on the basilar membrane in the cochlea, is composed of thousands of delicate hair cells (auditory sensory cells) and supporting cells [79]. The organ of Corti contains two classes of hair cells, inner hair cells (IHCs) contacting the majority of the afferent nerve fiber and outer hair cells (OHCs) which play a role in amplifying the mechanical stimulus and have the majority of efferent innervations [80]. The function of IHCs is relaying acoustic information via multiple ribbon synapses that transmit rapidly without exhaust. OHCs are important for amplifying sound-induced vibrations. The amplification mechanism primarily involves contraction of outer hair cells, which are driven by changes in membrane potential and mediated by the protein prestin [80]. The cochlea also plays the role of a spectrum analyzer in which different sound frequencies are separated along the cochlea, with each hair cell being tuned to a narrow frequency range; amplification sharpens the frequency resolution and augments sensitivity 100-fold around the cell’s characteristic frequency. Genetic mutations and environmental factors such as acoustic overstimulation cause hearing loss through irreversible damage to the hair cell or degeneration of the inner cell system [78]. The stereociliary (hair) bundle, projecting from the top face of hair cells, is the organelle through which all mechanical stimuli are focused for detection by the transduction machinery. The hair cells in the organ of Corti can be destroyed by various factors, e.g., aging, loud noise, ototoxic chemicals, and ototoxic medications. Among these factors, exposure to loud noise is the most common cause of irreversible injury to hair cells, causing permanent sensorineural hearing loss [81].

ONIHL is an insidious illness and may progress until it advances to hearing disability [6]. Exposure to loud noise can result in a temporary threshold shift (TTS) and/or a permanent threshold shift (PTS) [82]. Continued exposure to excessive noise may lead to impaired transmission of both low- and high-frequency sounds to the brain [80, 82]. Furthermore, the cochlear blood flow may be poor; hair cells bear stereocilia on the apical surface and these can become a fused, splayed, or missing stereociliary bundle arrangement after significant noise exposure, hair cells and supporting structures disrupt hearing function; and ultimately, even nerve fibers that innervate hair cells disappear [83–86]. Changes in the stria vascularis are likely to decrease endocochlear function, thus decreasing the cochlear amplifying function for auditory signals and increasing the auditory threshold [87]. Along with the degeneration of cochlear nerve fibers, there is simultaneous degeneration within the central nervous system [30]. Because hair cells in mammalian species do not regenerate, once hair cells are destroyed, ONIHL is present permanently, regardless of the pathway of hair cell destructions [85].

Pure tone audiometric testing is used to detect and quantify the degree of ONIHL [88]. This provides a subjective measurement of hearing loss in individuals exposed to occupational noise and requires a voluntary response on the part of the person being tested [25]. The main characteristics of ONIHL are that most noise exposures are symmetric and display typical signs of notching at high frequencies of 3000, 4000, or 6000 Hz with recovery at 8000 Hz in audiogram testing [6, 89]. This notch presents at one of these frequencies and does not actually influence neighboring frequencies but once a notch occurs, additional frequencies may demonstrate notches, and the prominence of this notch may be affected by age-related hearing loss [89]. Therefore, ONIHL needs to be differentiated from age-related hearing loss in older persons.

Typically, ONIHL is bilateral and symmetric. However, there may be some atypically asymmetric presentation in hearing loss, particularly if there is a different degree of exposure to sound between the two ears. When a discrepancy is present, ONIHL is commonly more severe in the left ear, although the reasons for this phenomenon are still unclear [4]. It is estimated that approximately 90% of the world population is right-handed [88]. Some arguments have been raised that a person with right-handedness may be more likely to leave the left ear turned toward a noise-source from machine engine [41]. Similarly, for those who shoot firearms or play musical instruments, more severe hearing impairment is noted in the ear closest to the gun barrel or to one’s location within the band or orchestra, and the opposite ear receives relatively lower amplitude of sound due to the acoustic shadow effect [26, 41].

Recently, another theory of oxidative stress has been proposed to play a possible role in the pathogenesis of ONIHL [84, 90]. Reactive oxygen species (ROS) are a normal byproduct of cellular respiration, and a certain level of intracellular ROS is necessary for various cellular processes. However, excessive loads of ROS cause oxidative damage to DNA, lipids, and proteins and result in cell death [91, 92]. Previous studies have shown that noise exposure increases the levels of ROS in the cochlea, following by activation of signaling pathways leading to cell death [93, 94]. Although ROS play a role in the cascade of ONIHL, the mechanism by which noise generates these free radicals is still unknown [94]. Based on these hypotheses, some antioxidant compounds, e.g., N-acetyl-L-cysteine and D-methionine, are undergoing clinical trials for their use as otoprotective agents [95–101]. However, these agents are still in the animal study stage. More research is needed in the future.

In addition to continuously high levels of noise, short bursts of loud sound may also result in ONIHL [24]. Short bursts of loud sounds lead to cochlear hair cell injury and injury to the inner ear surrounding supporting cells, resulting in the degeneration of auditory nerve fibers [83]. A previous study indicated that noise exposure of at least 130 dB sound pressure level (SPL) is required to cause direct mechanical damage to the auditory system [93]. The level of inner ear cell injury and associated hearing loss is correlated with the intensity and duration of noise exposure [4, 102, 103]. Current data suggest that even sublethal injury of inner ear cells may accelerate the process of age-related hearing loss [102–104].

In addition, some animal studies have shown that noise exposure can cause injury at the synapse between the inner hair cells and auditory neurons that is not reflected on audiograms [105, 106]. It has been suggested that this synaptic injury may be related to functional hearing loss and the degradation of speech intelligibility among other noise in the presence of normal audiometric thresholds [106, 107]. The theory of synaptic injury has only been confirmed in animal models, and more evidence is needed to support this theory.

Except the direct damage on the auditory system, noise also can cause psychological and physiological disorders. Tinnitus, the subjective sensation of sound, is an effect of noise exposure that can be even more bothersome for individuals than hearing loss. Tinnitus often serves as an early sign of auditory injury [108]. The severity of tinnitus may be associated with the degree of NIHL. Tinnitus has several etiologies other than noise exposure. However, once hearing loss is noted, the cause of hearing loss is recognized as the cause of tinnitus, unless more evidence proves otherwise [107].

### Prevention measures

At present, ONIHL is an irreversible disease with no effective treatment [18]. Prevention remains the best option for limiting the deteriorations of hearing power. A safe and healthy work environment is a basic requirement for all workers. The principal purpose of prevention measures for ONIHL includes monitoring occupational noise exposures (e.g., periodic noise exposure monitoring), reducing noise exposure in workplaces (e.g., engineering controls, administrative controls, and personal hearing protection), and early detection before permanent damage to the inner ear (e.g., routine audiometric examinations and health education) [18, 108, 109].

For industrial noise, elimination or reduction of noise through engineering or administrative control is the best way of ONIHL intervention [4, 26]. The risk of ONIHL can be minimized if noise is decreased to below 80 dBA (weighted decibel relative to human ear) [21, 22]. For protection from hearing loss among workers, many nations have implemented legal standards regarding occupational noise exposure, and the majority (> 80%) of nations use a PEL of 85 dBA and a 3-dB exchange rate in workplaces [24, 106]. Engineering or scheduling changes is a very effective way to reduce the sources of noise in the workplace [110]. However, elimination of ONIHL is a large and long-term challenge at the individual, organizational policy, and population levels. The control measures for ONIHL need evidence-based evaluations that can provide policymakers with contextual evidence to implement ONIHL prevention and control programs.

Although decreasing noise production and personal exposure to noise through engineering and administrative controls may provide the most effective measures for reducing noise exposure of workers, these strategies are often difficult to achieve [103, 104]. When we cannot reduce the on-the-job environmental noise levels to acceptable standards, providing appropriate personal hearing protective devices (HPDs) and instructing workers to use protective devices become important alternative protective strategies [24, 110, 111].

Hearing protection is a secondary level of protection measure. Both earmuffs and earplugs are commonly used as personal HPDs among workers. Previous studies suggest that using personal HPDs is effective in the prevention of ONIHL [24, 100]. Data have shown that earplugs may not provide the advertised level of protection if employees are not instructed on their proper use [5]. Infrequent use of HPD and low perception of sound during HPD use are the most important factors influencing the effect of personal HPD usage in preventing ONIHL [112–116]. Continuous education of workers on the consistent use of HPD in noisy workplaces and implement of different interventional strategies are needed for promoting the use of HPD in the future.

## Conclusions

In summary, ONIHL is still the most prevalent occupational disease in the world. The findings from this review provide guidance to policymakers in terms of where resources might best be used and can provide insight into the effectiveness of other past interventions. Treatment strategies are still in the developmental stages; before they become universally available, the main strategy for reducing the prevalence of ONIHL is prevention.

## Data Availability

Data sharing is not applicable to this article as no dataset was generated or analyzed during the current study.

## Abbreviations

AFFH: Agriculture, forestry, fishing, hunting
DALY: Disability-adjusted life year
HCA: Hearing Conservation Amendment
HLPP: Hearing loss prevention program
HPDs: Hearing protective devices
IHCs: Inner hair cells
MT: Mechanotransducer
NIOSH: National Institute for Occupational Safety and Health
NIHL: Noise-induced hearing loss
ONIHL: Occupational noise-induced hearing loss
OSHA: Occupational Safety and Health Administration
OGE: Oil and gas extraction
OHCs: Outer hair cells
PEL: Permissible exposure limit
REL: Recommended exposure limit
SPL: Sound pressure level
TTS: Temporary threshold shift
TWA: Time-weighted average
